# Cost analysis of pre-pectoral implant-based breast reconstruction

**DOI:** 10.1038/s41598-022-21675-6

**Published:** 2022-10-20

**Authors:** Sachin Chinta, Daniel J. Koh, Nikhil Sobti, Kathryn Packowski, Nikki Rosado, William Austen, Rachel B. Jimenez, Michelle Specht, Eric C. Liao

**Affiliations:** 1 Boston University School of Medicine, Boston, MA USA; 2 Department of Plastic and Reconstructive Surgery, The Warren Alpert Medical School of Brown University, Providence, RI USA; 3 Division of Plastic and Reconstructive Surgery, Massachusetts General Hospital, 15 Parkman Street, WACC 435, Boston, MA 02114 USA; 4 Division of Radiation Oncology, Massachusetts General Hospital, Boston, MA USA; 5 Division of Surgical Oncology, Massachusetts General Hospital, Boston, MA USA

**Keywords:** Breast cancer, Medical research, Outcomes research, Cancer, Health care, Health care economics

## Abstract

With improvement in mastectomy skin flap viability and increasing recognition of animation deformity following sub-pectoral implant placement, there has been a transition toward pre-pectoral breast reconstruction. While studies have explored the cost effectiveness of implant-based breast reconstruction, few investigations have evaluated cost with respect to pre-pectoral versus sub-pectoral breast reconstruction. A retrospective review of 548 patients who underwent mastectomy and implant-based breast reconstruction was performed from 2017 to 2020. The demographic and surgical characteristics of the pre-pectoral and sub-pectoral cohorts were well matched, except for reconstructive staging, as patients who underwent pre-pectoral reconstruction were more likely to undergo single-stage instead of two-stage reconstruction. Comparison of institutional cost ratios by reconstructive technique revealed that the sub-pectoral approach was more costly (1.70 ± 0.44 vs 1.58 ± 0.31, p < 0.01). However, further stratification by laterality and reconstructive staging failed to demonstrate difference in cost by reconstructive technique. These results were confirmed by multivariable linear regression, which did not reveal reconstructive technique to be an independent variable for cost. This study suggests that pre-pectoral breast reconstruction is a cost-effective alternative to sub-pectoral breast reconstruction and may confer cost benefit, as it is more strongly associated with direct-to-implant breast reconstruction.

## Introduction

Implant-based breast reconstruction is the most common method of breast reconstruction world-wide^1–5^. Over the past two decades, breast implants have routinely been placed underneath the pectoralis major muscle. Often sub-pectoral reconstruction is carried out in a staged fashion, where tissue expanders are first placed beneath the pectoralis major muscle to allow for coverage of the upper pole of the device, with the inferior margin either exposed to the subcutaneous mastectomy flap or supported by surgical material along the inferolateral aspect^6–11^. Recently, implant-based breast reconstruction has transitioned toward direct prosthesis placement at the time of mastectomy, as the viability of mastectomy skin flaps continues to improve with advances in nipple-sparing mastectomy technique and careful patient selection^4,11–17^. With robust mastectomy skin flaps and increasing awareness of adverse sequelae of sub-pectoral reconstruction, there has been a shift toward implant placement in the subcutaneous or pre-pectoral plane^18–24^.

Despite its wide-spread use, sub-pectoral breast reconstruction poses various reconstructive challenges. In recent retrospective cohort studies, sub-pectoral implant placement has been associated with higher rates of explantation, post-operative pain, muscle spasm, capsular contracture, and animation deformity compared to pre-pectoral breast reconstruction^23–28^. Different factors must be considered when choosing pre-pectoral placement, chief among them are the mastectomy skin flap thickness and viability^22,29^. As surgical oncologists more consistently preserve vascularity of mastectomy skin flaps, pre-pectoral placement of implants or tissue expanders has been increasingly utilized to restore breast form following mastectomy at many high-volume centers^19,29–34^. Recent retrospective studies and meta-analyses have found pre-pectoral reconstruction to demonstrate a favorable or comparable safety profile when compared to sub-pectoral reconstruction. In fact, pre-pectoral reconstruction often demonstrates lower rates of capsular contracture and skin flap necrosis^19,23,25,35–38^. However, pre-pectoral breast reconstruction also has limitations. Several studies have demonstrated increased rates of implant rippling following pre-pectoral breast reconstruction, often requiring fat grafting and implants with higher fill ratios, greater gel cohesivity, or thicker shells^38–46^. This combination of factors may contribute to increased cost associated with pre-pectoral breast reconstruction. Furthermore, pre-pectoral implant placement often requires a greater number of acellular dermal matrix (ADM) or synthetic mesh sheets to provide soft tissue support, which has led to concern about associated costs.

Whereas numerous studies have investigated the cost-effectiveness of implant-based breast reconstruction, few have evaluated cost in the setting of reconstructive plane. In this study, we assessed the mean cost differences between patients who underwent pre-pectoral versus sub-pectoral breast reconstruction. Additionally, we examined differences in complication rates and revision procedures. We hypothesized that while there are multiple factors, such as operating room (OR) costs and surgical adjunct usage, that may increase or decrease cost for pre-pectoral breast reconstruction, it is feasible to achieve excellent patient outcomes and be cost effective using a pre-pectoral breast reconstruction approach.

## Methods

### Study design and population

Institutional review board approved retrospective chart review was conducted at Massachusetts General Hospital. Patients who underwent one- and two-stage implant-based breast reconstruction following oncologic management of breast cancer or prophylactic mastectomy between January 2017 and December 2020 were identified, yielding a cohort of 614 patients. This time period was selected as the experience of pre-pectoral breast reconstruction began in our institution in 2015, with the learning curve stabilizing by 2017^23,25,47^. To minimize patient selection bias, only cases performed by surgeons with experience in pre-pectoral breast reconstruction were included (over 50 cases per year). A minimum threshold of 50 cases was used, as multiple papers in plastic surgery have shown that to be the inflection point for procedural learning curves^48,49^. Those who underwent autologous flap-based reconstruction, abdominoplasty, or concurrent elective aesthetic procedures were excluded. Sixty-six cases were excluded.

Patient characteristics, tumor pathology, and surgical characteristics were recorded. All patients included in this study were followed up for an average of 10.33 months.

### Surgical technique

Surgical technique of sub-pectoral breast reconstruction utilized ADM or Vicryl mesh to support the mastectomy flap in the inferolateral aspect^50^. Pre-pectoral breast reconstruction utilized ADM, Vicryl, or a combination in the inferolateral aspect of the mastectomy flap or around the entire implant^23,25,51,52^.

### Outcome analysis

Additional procedures performed after the initial operation were classified into two distinct groups: re-operation due to immediate complications and elective revision. The average number of additional procedures and percentage of patients undergoing subsequent procedures were noted. Incidence of both immediate and delayed complications were recorded.

Elective revisions addressed delayed cosmetic complications through capsule, symmetry, implant exchange, and fat grafting procedures.

### Statistical analysis

The dataset was analyzed using SPSS 25 (IBM Corp., Armonk, NY). Statistical significance was defined as *p* < 0.05. Demographic, oncologic, surgical, complication, and revision data were analyzed either via two tailed t-tests for continuous variables or via chi-square test for non-continuous variables. Assumptions for homogeneity and normality were confirmed via the Levene’s and Shapiro–Wilk test, respectively. If the data failed these tests, equal variances were not assumed. Independent two tailed t-tests with 95% confidence intervals were used to determine statistical significance between the sub- and pre-pectoral groups for all other continuous variables.

A multivariable linear regression was used to evaluate the effect of covariates on overall cost ratio. The following covariates were included: surgical approach, obesity, laterality, reconstructive staging, chemotherapy, and radiation. These covariates were chosen based on studies demonstrating known risk-factors of post-operative complication in breast reconstruction^53^. These predictors of adverse outcome were controlled for, as they could contribute to a higher rate of revision surgery and increase cost.

### Cost analysis

This study used previously established techniques for cost-minimization analysis in which the costs associated with sub- and pre-pectoral breast reconstruction were compared^1^. Additional costs associated with revision procedures were not included. This analysis obtained cost data at the hospital-level rather than the patient-level, as patient charge data can vary with differences in insurance reimbursement.

The cost associated with ADM, Vicryl mesh, implant, and tissue expander were set as fixed estimates, as different manufacturers have materials of varying costs, which are subject to variability across hospital facilities due to contracting agreements. This cost analysis used estimated hospital costs of $3500/unit of ADM, $550/unit of Vicryl mesh, $750/implant, and $1100/tissue expander which represented the average cost of each device across manufacturers, device subtypes, and facilities.

All cost values were divided by the mean dollar amount of the least costly procedure (unilateral direct-to-implant reconstruction), resulting in a normalized cost ratio^1^. The costs obtained included hospital fees (staff payment, OR costs, in-patient costs) as well as actual supply costs (suture material, etc.). Using this hospital data, a base institutional cost was determined for 3 CPT codes (insertion of breast implant, tissue expander placement, and tissue expander removal). From these base costs, the cost of breast implants, surgical adjuncts, and OR costs/min were added to determine the final cost. The base costs were combined dependent on the staging of reconstruction. Length of stay was not included in the final cost, as a portion of our data was collected during the COVID-19 pandemic where our institution initiated a same-day mastectomy/reconstruction outpatient protocol.

### Ethics declarations

All procedures performed in studies involving human participants were in accordance with the ethical standards of the Massachusetts General Hospital Institutional Review Boards, the national research committee, and with the 1964 Helsinki declaration and its later amendments or comparable ethical standards. This study did not receive any external funding.

#### Ethics approval

 This paper does not contain studies with any animals performed by any of the authors.

#### Informed consent

 This study was conducted with a waiver of informed consent as study protocol was retrospective and did not deviate from the standard of care. All procedures and data collection was done at Massachusetts General Hospital and is compliant with Massachusetts General Hospital IRB and ethical guidelines. Consent was waived by the IRB of Massachusetts General Hospital.

## Results

### Patient characteristics

**Figure 1:**
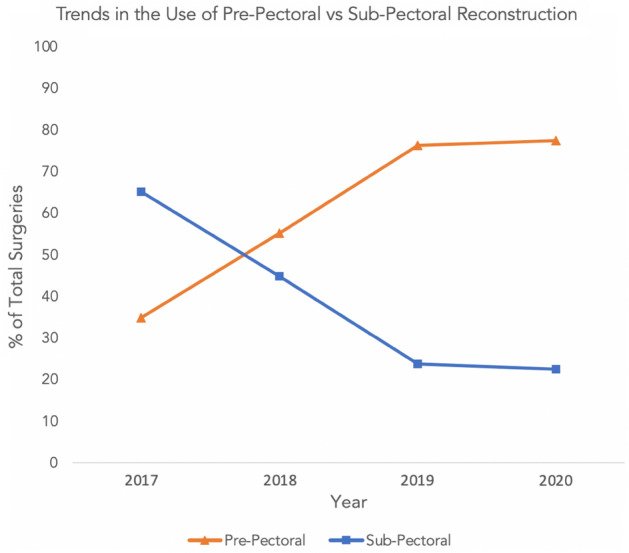
Trends in utilization of pre-pectoral implant-based breast reconstruction as a percentage of total reconstruction procedures performed within a single year (based on full patient cohort, n = 548). This figure shows the trends in utilization of pre-pectoral and sub-pectoral breast reconstruction from the years of 2017 to 2020. The inflection transition point of the two reconstructive techniques occurs between the years of 2017 and 2018.

The pre-pectoral and sub-pectoral breast reconstruction cohorts were well matched, as 58.6% of patients underwent pre-pectoral breast reconstruction and 41.1% of patients underwent sub-pectoral breast reconstruction. Our dataset showed progressive increase in the number of pre-pectoral cases and concomitant decrease in sub-pectoral cases, with the inflection transition point between 2017 and 2018 (Fig. 1). The patient demographics and comorbidities of the two cohorts were comparable (Table 1).

**Table 1:** Patient demographics and oncologic characteristics.

Variable	Total (%)	Pre-pectoral (%)	Sub-pectoral (%)	*p*
No. of patients	548	321 (58.6)	227 (41.4)	
Mean age at surgery (year)		50.83 ± 10.62	50.32 ± 10.84	0.587
**Mean BMI (kg/m**^**2**^**)**		26.95 ± 8.89	25.84 ± 5.15	0.092
Obesity	114 (20.8)	67 (20.9)	47 (20.7)	0.962
Diabetes mellitus I/II	22 (4.0)	11 (3.4)	11 (4.8)	0.405
Active smoking	30 (5.5)	17 (5.3)	13 (5.7)	0.827
Prior radiation	47 (8.6)	24 (7.5)	23 (10.1)	0.274
PMRT	125 (22.8)	73 (22.7)	52 (22.9)	0.964
Neoadjuvant chemotherapy	109 (19.9)	66 (20.6)	43 (18.9)	0.640
Adjuvant chemotherapy	172 (31.4)	99 (30.8)	73 (32.2)	0.743
BRCA 1/2 positive	85 (15.5)	47 (14.6)	38 (16.7)	0.504
**Pathology**				
Invasive ductal carcinoma	355 (64.8)	220 (68.5)	135 (59.5)	0.029*
Invasive lobular carcinoma	173 (31.6)	117 (36.4)	56 (24.7)	0.003*
Ductal carcinoma in-situ	413 (75.4)	251 (78.2)	162 (71.4)	0.068
Lobular carcinoma in-situ	196 (35.8)	128 (39.9)	68 (30.0)	0.017*

This table compares the demographics and oncologic characteristics of patients who underwent sub-pectoral reconstruction versus patients who underwent pre-pectoral reconstruction in 2017 through 2020. The two cohorts of patients significantly differ in the incidence of invasive carcinomas and lobular carcinoma in-situ. Continuous variables were analyzed via independent two-tailed t-tests (95% confidence interval) and displayed with standard deviation values. Non-continuous variables were analyzed via Pearson’s chi square.

*BMI* body mass index, *PMRT* post-mastectomy radiation therapy, *BRCA* breast cancer gene; ± SD; 95% CI.

Obesity defined as BMI ≥ 30.

*Statistically significant (*p* < 0.05).

Outside of ductal carcinoma in situ, tumor characteristics differed between the sub-pectoral and the pre-pectoral groups. Rates of invasive ductal carcinoma and invasive lobular carcinoma were greater in the pre-pectoral cohort (68.5% v 59.5%, *p* < 0.03 and 36.4% v 24.7%, *p* < 0.003). The higher incidence of invasive cancers in the pre-pectoral cohort was expected as invasive cancer has been shown to be a significant predictor of post-mastectomy radiation therapy (PMRT), and patients expected to undergo PMRT are routinely offered pre-pectoral breast reconstruction to mitigate radiation associated capsular contracture at our institution^25,54–56^.

The surgical characteristics between the sub-pectoral and pre-pectoral cohorts differed in terms of reconstruction staging (Table 2). While the majority of sub-pectoral and pre-pectoral reconstructions were carried out in a single stage approach, the pre-pectoral group had a greater percentage of direct-to-implant (DTI) reconstruction procedures than sub-pectoral group (91.6% vs 79.3%, *p* < 0.001).

**Table 2:** Surgical characteristics.

Variable	Total (%)	Pre-pectoral (%)	Sub-pectoral (%)	*p*
**Laterality**				0.201
Unilateral	208 (38.0)	129 (40.2)	79 (34.8)	
Bilateral	340 (62.0)	192 (59.8)	148 (65.2)	
**Indication**				0.446
No. of prophylactic procedures	45 (8.2)	25 (7.8)	20 (8.8)	
No. of therapeutic procedures	503 (91.8)	296 (92.2)	207 (91.2)	
**Type of mastectomy**				0.446
Skin-sparing	503 (91.8)	296 (92.2)	207 (91.2)	
Nipple-sparing	45 (8.2)	25 (7.8)	20 (8.8)	
**Staging**				0.000*
Direct-to-implant reconstruction	474 (86.5)	294 (91.6)	180 (79.3)	
Staged tissue-expander reconstruction	74 (13.5)	27 (8.4)	47 (20.7)	
Mean implant size (*mL*)		457.7 ± 174.8	444.4 ± 201.5	0.410

This table analyzes the surgical characteristics of the pre-pectoral implant group and the sub-pectoral implant group from the years of 2017 through 2020. The two cohorts differ significantly in terms of reconstructive staging. Continuous variables were analyzed via independent two-tailed t-tests (95% confidence interval) and displayed with standard deviation values. Non-continuous variables were analyzed via Pearson’s chi square.

ADM, acellular dermal matrix; ± SD; 95% CI.

*Statistically significant (*p* < 0.05).

### Complication and revision rates

With regard to post-operative complications, pre-pectoral and sub-pectoral implant placement groups differed in rates of delayed complications (Table 3). The pre-pectoral group demonstrated significantly higher rates of visible implant rippling (8.7% vs 4.4%, *p* = 0.05). The sub-pectoral group had a greater incidence of both capsular contracture and animation deformity (12.8% vs 4.4%, *p* < 0.001 and 2.6% vs 0.0%, *p* < 0.004)^23,25^.

**Table 3:** Summary of Outcomes.

Variable	Total (%)	Pre-pectoral (%)	Sub-pectoral (%)	*p*
**Immediate complications**				
Seroma	41 (7.5)	23 (7.2)	18 (7.9)	0.738
Hematoma	18 (3.3)	9 (2.8)	9 (4.0)	0.453
Skin necrosis	37 (6.8)	21 (6.5)	16 (7.0)	0.816
Infection	31 (5.7)	20 (6.2)	11 (4.8)	0.489
Explant	38 (6.9)	21 (6.5)	17 (7.5)	0.667
**Delayed complications**				
Capsular contracture	43 (7.8)	14 (4.4)	29 (12.8)	0.000*
Animation deformity	6 (1.1)	0 (0.0)	6 (2.6)	0.003*
Rippling	38 (6.9)	28 (8.7)	10 (4.4)	0.050

This table examines the surgical outcomes of the index surgery of the both the pre-pectoral implant group and the sub-pectoral implant group. The delayed complications (capsular contracture, animation deformity, and rippling) are the only surgical outcomes that significantly differ between the two cohorts. Continuous variables were analyzed via independent two-tailed t-tests (95% confidence interval) and displayed with standard deviation values. Non-continuous variables were analyzed via Pearson’s chi square.

95% CI.

*Statistically significant (*p* < 0.05).

Patients in the pre-pectoral cohort were less likely to undergo a subsequent procedure (27.1% vs 39.6%, *p* < 0.003), and were found to have fewer numbers of revision procedures per patient (0.36 ± 0.69 procedures vs 0.68 ± 1.08 procedures, *p* < 0.001) (Table 4). There was no significant difference between in re-operations due to acute complications between the two cohorts. Thus, the difference seen in subsequent procedures was attributed to a difference in the number of elective revisions in the sub-pectoral cohort (28.2% vs 15.5%, *p* < 0.001).

**Table 4:** Revision procedure data.

Variable	Total (%)	Pre-pectoral (%)	Sub-pectoral (%)	*p*
Average no. of additional procedures	0.49 ± 0.89	0.36 ± 0.69	0.68 ± 1.08	0.000*
Additional procedures	177 (32.3)	87 (27.1)	90 (39.6)	0.002*
Re-operation due to IC	63 (11.5)	37 (11.5)	26 (11.5)	0.979
Elective revisions	114 (20.6)	50 (15.5)	64 (28.2)	0.000*
Fat grafting	32 (5.8)	13 (4.0)	19 (8.4)	0.034*

This table analyzes follow-up procedure data of both the pre-pectoral implant cohort and the sub-pectoral implant cohort. The average number and frequency of additional procedures significantly differs between the sub-pectoral and the pre-pectoral groups. Additionally, the sub-pectoral cohort was found to have a higher incidence of elective procedures. Continuous variables were analyzed via independent two-tailed t-tests (95% confidence interval) and displayed with standard deviation values. Non-continuous variables were analyzed via Pearson’s chi square.

± SD; 95% CI.

First row values are an average of revisions across all patients in each group. (e.g., 0.36 revision procedures per patient undergoing pre-pectoral reconstruction).

Additional Procedures: percentage patients per cohort that underwent subsequent procedure either for acute complication or elective revision.

IC, immediate complication; includes seroma, hematoma, skin flap necrosis, infection, and explantation.

Elective revisions include fat grafting, capsule procedures, symmetry procedures, and implant exchange.

*Statistically significant (*p* < 0.05).

### Cost analysis

A whole group cost analysis was first conducted between cohorts. The pre-pectoral group was found to have a lower mean cost ratio than the sub-pectoral group (1.58 ± 0.31 vs 1.70 ± 0.44, *p* < 0.001) (Table 5). The pre-pectoral approach was found to have higher average ADM usage (0.90 ± 0.44 sheets vs 0.72 ± 0.48 sheets, *p* < 0.02) and higher Vicryl mesh usage (0.94 ± 0.30 sheets vs 0.30 ± 0.47 sheets, *p* < 0.001, respectively). Furthermore, the pre-pectoral group had shorter OR times (139.90 ± 44.13 min vs 172.83 ± 34.66 min, *p* < 0.001).

**Table 5:** Overall cohort mean cost comparison.

Variable	Pre-pectoral	Sub-pectoral	*p*
No. of patients	321	227	
Mean cost ratio	1.58 ± 0.31	1.70 ± 0.44	0.000*
OR time^70^	139.90 ± 44.13	172.83 ± 34.66	0.000*
ADM usage (*sheets*)	0.90 ± 0.44	0.72 ± 0.48	0.014*
Vicryl usage (*sheets*)	0.94 ± 0.30	0.30 ± 0.47	0.000*

This table describes the mean cost comparison of the sub-pectoral and pre-pectoral cohorts as a whole group. The pre-pectoral group was found to utilize significantly more ADM and Vicryl mesh while having lower operative time. The sub-pectoral approach to breast reconstruction was found to be more costly. Continuous variables were analyzed via independent two-tailed t-tests (95% confidence interval) and displayed with standard deviation values.

± SD; 95% CI.

Cost ratio: a given cost value divided by the base procedural cost of unilateral direct-to-implant reconstruction.

Values for ADM (acellular dermal matrix) or Vicryl mesh are averages across all patients in each group (e.g., 0.90 sheets of ADM used per patient in the pre-pectoral cohort).

Operating room (OR) time is measured as procedure start to end time in minutes.

*Statistically significant (*p* < 0.05).

The cohorts were then divided based upon laterality and staging of reconstruction (Table 6). While these sub-groups demonstrated no difference in cost ratio between the pre-pectoral and sub-pectoral cohorts, they varied in other measured variables. Pre-pectoral reconstruction was associated with significantly shorter OR times and greater surgical adjunct use for both unilateral and bilateral DTI sub-groups. The unilateral two-stage tissue expander sub-group differed only in the number of Vicryl mesh used between the pre- and sub-pectoral groups (0.64 ± 0.50 sheets vs 0.15 ± 0.38 sheets, *p* < 0.01, respectively). Multivariable linear regression demonstrated that both laterality and reconstruction staging have an independent association with the cost ratio (adjusted R^2^ = 0.871; β = 0.266, *p* < 0.001 and β = 0.897, *p* < 0.001, respectively) (Table 7).

**Table 6:** Sub-group analysis by laterality and reconstruction staging.

Variable	Pre-pectoral	Sub-pectoral	*p*
**Unilateral DTI**			
No. of patients	115	65	
Mean cost ratio	1.37 ± 0.10	1.37 ± 0.08	0.752
OR time^70^	139.90 ± 44.13	172.83 ± 34.66	0.000*
ADM usage (*sheets*)	0.90 ± 0.44	0.72 ± 0.48	0.014*
Vicryl usage (*sheets*)	0.94 ± 0.30	0.30 ± 0.47	0.000*
**Unilateral TE**			
No. of patients	14	13	
Mean cost ratio	2.34 ± 0.07	2.39 ± 0.11	0.155
OR time^70^	158.57 ± 62.16	188.08 ± 51.72	0.194
ADM usage (*sheets*)	0.71 ± 0.61	0.85 ± 0.55	0.563
Vicryl usage (*sheets*)	0.64 ± 0.50	0.15 ± 0.38	0.008*
**Bilateral DTI**			
No. of patients	179	115	
Mean cost ratio	1.59 ± 0.15	1.56 ± 0.15	0.196
OR time^70^	186.23 ± 55.38	223.52 ± 46.73	0.000*
ADM usage (*sheets*)	1.61 ± 0.93	1.36 ± 0.91	0.025*
Vicryl usage (*sheets*)	1.73 ± 0.68	0.55 ± 0.84	0.000*
**Bilateral TE**			
No. of patients	13	34	
Mean cost ratio	2.56 ± 0.12	2.55 ± 0.19	0.835
OR time^70^	241.23 ± 70.98	272.12 ± 72.06	0.163
ADM usage (*sheets*)	1.23 ± 1.01	1.03 ± 0.98	0.540
Vicryl usage (*sheets*)	1.00 ± 1.00	0.51 ± 0.83	0.100

This table further describes the cost differences between sub-pectoral and pre-pectoral breast reconstruction by conducting analysis independent of both laterality and staging. None of the sub-group analyses found significant cost differences between sub-pectoral and pre-pectoral breast reconstruction. While differences in ADM usage, operative time, and Vicryl mesh usage remained in some of the sub-group comparisons. Continuous variables were analyzed via independent two-tailed t-tests (95% confidence interval) and displayed with standard deviation values.

Cost ratio: a given cost value divided by the base procedural cost of unilateral direct-to-implant reconstruction.

*DTI* direct-to-implant, *TE* tissue expander; ± SD; 95% CI.

Values for ADM (acellular dermal matrix) or Vicryl mesh are averages across all patients in each group.

OR time is measured as procedure start to end time in minutes.

*Statistically significant (*p* < 0.05).

**Table 7:** Multivariable linear regression analysis of cost ratio.

Covariate	Standardized coefficient beta	Lower CI	Upper CI	t	*p*
Surgical technique	0.017	− 0.010	0.037	1.11	0.267
Obesity	0.022	− 0.007	0.048	1.46	0.146
PMRT	0.013	− 0.020	0.044	0.72	0.469
Chemotherapy	0.015	− 0.015	0.038	0.83	0.406
DTI v. TE	0.897	0.951	1.018	57.26	0.000*
Laterality	0.266	0.182	0.229	17.27	0.000*

This table shows a multivariable linear regression based on covariates that are known risk-factors of post-operative complications in breast reconstruction. The regression found independent associations of both laterality and reconstructive staging with cost ratios. The regression utilized a confidence interval of 95% and demonstrated strong linearity with an adjusted R square value of 0.871.

*Statistically significant (*p* < 0.05).

*PMRT* post-mastectomy radiation therapy, *DTI* direct-to-implant, *TE* tissue expander, *CI* confidence interval (95%).

Adjusted R^2^ = 0.871; Standard Error of the Estimate = 0.135.

Surgical technique defined as sub- and pre-pectoral breast reconstruction.

Chemotherapy included both neoadjuvant and adjuvant therapy.

Laterality included unilateral and bilateral breast reconstruction.

## Discussion

Subcutaneous implant placement has recently emerged as a viable alternative to submuscular implant placement. Studies suggest that pre-pectoral implant-based breast reconstruction mitigates the risk of animation deformity, muscle dysfunction, and capsular contracture, while also providing patients with aesthetic and functional benefits following mastectomy^4,11,12,18,19,57^. While many studies have compared post-operative complication rates between sub-pectoral and pre-pectoral reconstruction, few have analyzed long-term economic impact between the two types of reconstruction^58–60^.

This study found pre-pectoral breast reconstruction to be more cost-effective at our institution. This supports our hypothesis that pre-pectoral breast reconstruction is a cost-effective option when compared to traditional sub-pectoral methods. On a whole group level, the pre-pectoral cohort cost ratio was significantly lower than that of the sub-pectoral cohort, despite being associated with greater surgical adjunct utilization. Our data suggests that cost saving was primarily realized due to decreased operative time and differences in operative characteristics, such that sub-group analyses were conducted to further parse out the basis of this cost difference.

The unilateral and bilateral DTI sub-group analyses yielded similar findings when compared to the whole group analysis, with the pre-pectoral approach having significantly shorter average operative time and greater surgical adjunct use. However, there was little difference in cost ratio between the sub- and pre-pectoral groups. This suggests that the increase in surgical adjunct cost was offset by the decrease in operating room cost. Thus, the difference in cost observed in whole-group univariate analysis was likely influenced by covariates, more specifically, laterality and staging. In controlling for potential confounders in a multivariable linear regression analysis, there was no independent association between surgical approach (sub- and pre-pectoral) and cost, but the analysis demonstrated that both laterality and staging of reconstruction independently affected cost ratios (Table 7). This observation coupled with the fact that pre-pectoral reconstruction was more commonly associated with DTI reconstruction, suggests that the preponderance of DTI reconstruction within the pre-pectoral cohort may be a primary factor in the associated cost savings. Whereas costs incurred due to additional usage of ADM and Vicryl mesh may be offset by cost saved due to less operating room time, the difference between performing a DTI procedure versus a staged operation was sufficient to significantly reduce the cost ratio for the pre-pectoral cohort overall. It is important to highlight that ADM usage is dependent on the method of pre-pectoral breast reconstruction. In this study, the most common method of pre-pectoral reconstruction required two ADM sheets per breast; however, other reconstructive methods require 3–4 sheets of ADM per breast as the whole implant is wrapped. If the study had been done primarily through these other methods, the increased ADM usage may negate the cost savings shown in this study^51,52,61,62^.

Analysis of the number and frequency of revision procedures undergone by patients provides further insight into cost savings between pre- and sub-pectoral breast reconstruction. While the number of patients undergoing revision procedures due to acute complications was not significantly different between our cohorts, the sub-pectoral group had higher rates of revision overall (39.6% vs 27.1%) due to a higher rate of elective revision (28.2% vs 15.5%). Furthermore, the sub-pectoral cohort was found to have a greater incidence of fat grafting (8.4% vs 4.0%). Several other studies have reported higher rates of post-operative revision following sub-pectoral reconstruction, despite similar immediate complication safety profiles when compared to pre-pectoral reconstruction^23,42^. As revision procedure cost data was outside the scope of our study, it is difficult to ascertain the exact impact of the additional procedures on cost. However, it is reasonable to speculate that, despite there being no difference in cost following breast reconstruction, it is likely that the decreased need for revision procedures following pre-pectoral reconstruction could generate additional cost-saving over time.

In addition, our study investigated differences in rates of post-operative complication between pre-pectoral and sub-pectoral breast reconstruction, as immediate and delayed complication may provide insight into potential cost differences. Our univariate analysis demonstrated a similar safety profile between both reconstructive methods, supporting our initial hypothesis. Of note, there was a significantly higher rate of capsular contracture and animation deformity in the submuscular group (Table 3). These findings further support previous reports describing disruption of the overlying fascia and muscle during sub-pectoral reconstruction as factors that may contribute to increased rates of contracture, animation deformity, pain and muscle dysfunction^23–25,30^. Further analysis revealed that rippling was associated with pre-pectoral reconstruction. This result is consistent with previous literature that has demonstrated an increased rate of rippling, particularly in the upper pole, following pre-pectoral reconstruction due to issues with skin flap viability and vascularity^39–43,63^. Importantly, pre-operative patient selection is essential to ensure successful operative intervention and mitigate post-operative complication. While our institution did not use risk-assessment scores to evaluate patients prior to selection of reconstruction plane, central factors, such as skin flap viability, patient activity status, and functional goals, were considered in the shared decision making process between surgeon and patient^64^.

A preliminary report by Cattelani et al. examined a cohort of 86 patients, comparing functional outcomes and analyzing cost differences between pre-pectoral and sub-pectoral breast reconstruction. Pre-pectoral implant placement was associated with decreased post-operative pain, reduced time to functional recovery, and reduced financial burden^58^. Despite a well matched cohort, the authors emphasized the need for a larger cohort and a longitudinal analysis of complications. More recently, Viezel-Mathieu et al. reported a 25% reduction in cost when comparing direct-to-implant pre-pectoral reconstruction and two-stage sub-pectoral reconstruction^65^. However, by comparing single-stage reconstruction to two-stage reconstruction there is inherent cost saving that is attributable to number of operations. This renders assessing the specific financial benefit attributed to pre-pectoral reconstruction difficult. Our study aimed to address the current gaps by not only looking at long term complications and revision rates, but also by conducting large-scale cohort analysis that accounted for both laterality and staging of reconstruction.

Limitations of this study include its retrospective design, patient selection bias, and scope of application. While this study showed cost savings associated with the pre-pectoral approach, it cannot make conclusions about cost utility. In order to conduct valid cost-utility analysis, specific health benefits must be linked to cost savings, in the form of patient-reported outcomes or quality-adjusted life years^66–68^. Additionally, our data was limited for mastectomy specimen size. Differences in native breast size could account for longer operative times. However, this study used final implant size and BMI as effective surrogate markers, in which there were no differences between the two cohorts. Thus, we hypothesize that the decrease in operative time in the pre-pectoral cohort is related to time saved by avoiding submuscular dissection.

Because this study was conducted using patient data from a tertiary academic center, there may be a potential for selection bias, as our institution typically treats patients that require higher acuity or more complex oncological care. However, the study’s large sample size and well-matched pre- and sub-pectoral cohorts may help mitigate the potential effects of selection bias. Furthermore, there may be innate differences in operative technique and post-operative care between surgeons. To limit the impact of variable surgical technique and preference, the study included patients from multiple, experienced providers. Lastly, as costs were analyzed relative to reimbursement for a tertiary academic medical center within the United States, this study may not be generalized to actualized health costs from the patient perspective or other countries, due to varying insurance reimbursement rates, billing protocols, and differences in operative technique^69^.

Despite these limitations, the strength of this study lies in the detailed analysis of cost, complications, and revision procedure differences between a large cohort of patients, over a mean follow up time of 10.33 months (at the time of manuscript submission). This study represents one of the largest and most comprehensive cost studies to date exploring the hospital cost differences between pre-pectoral and sub-pectoral breast reconstruction. The main advantage of this study lies in the stratified analysis of the comparison cohorts. By dividing our data based on laterality and staging of reconstruction, we were able to identify key factors that influence cost differences between the two reconstructive methods.

## Conclusion

In this cost-minimization analysis comparing pre-pectoral and sub-pectoral breast reconstruction, we found that pre-pectoral breast reconstruction is a cost-effective alternative to submuscular reconstruction. Additional studies are necessary to better elucidate the cost burden at the patient-level, examine patient reported outcomes to ascertain cost utility, and to further guide shared clinical decision making for patients undergoing implant-based breast reconstruction.

## Data Availability

All data was acquired via retrospective chart review of over 1000 patients that have been treated by our team at MGH. The datasets generated and/or analyzed during the current study are not publicly available due identifiable patient information that would compromise individual privacy. Upon request, de-identified raw data would be generated for conditional access. All of this data will be available upon reasonable request from the corresponding author.
